# A platform for Bioengineering Tissue Membranes from cell spheroids

**DOI:** 10.1016/j.mtbio.2025.101526

**Published:** 2025-01-31

**Authors:** Quang Bach Le, Hariharan Ezhilarasu, Weng Wan Chan, Alok Tanala Patra, Priya Murugan, Shashaank Abhinav Venkatesh, Yean Kai Tay, Shin Ru Lim, Ahmad Amirul Abdul Rahim, Jia Sheng Zach Lee, Xuezhi Bi, Deepak Choudhury

**Affiliations:** a Biomanufacturing Technology (BMT), Bioprocessing Technology Institute (BTI), Agency for Science, Technology and Research (A∗STAR), 20 Biopolis Way, 138668, Singapore; b Department of Food Science and Technology, National University of Singapore, Singapore

**Keywords:** Biomanufacturing, Cell therapy, Tissue therapy, Tissue engineering, Bioengineering, Tissue membrane, Cell spheroids, Mesenchymal stem cells, MSCs, Cartilage, Platform technology

## Abstract

Cell spheroids are essential building blocks for engineering tissues like cartilage, bone, liver, cardiac, pancreatic, and neural tissues, but controlling their fusion and organisation is challenging. Spheroids tend to fuse into a larger mass, impeding nutrient and waste diffusion. To overcome this, we developed a method to assemble spheroids into a thin layer by using two mesh scaffolds to spread them evenly, and a solid frame with grid to secure the assembly. This allows the spheroids to fuse into a thin membrane-like tissue, allowing better medium diffusion during cell culture. We demonstrated this method by producing cartilage tissue membranes from human mesenchymal stem cell spheroids undergoing chondrogenic differentiation, evaluating spheroid sizes, assembly timing, fusion process and membrane thickness. Our method is a versatile platform for producing tissue membranes from cell spheroids, with significant potential in tissue engineering for creating functional tissue constructs from various cell types.

## Graphical abstract

**Figure:**
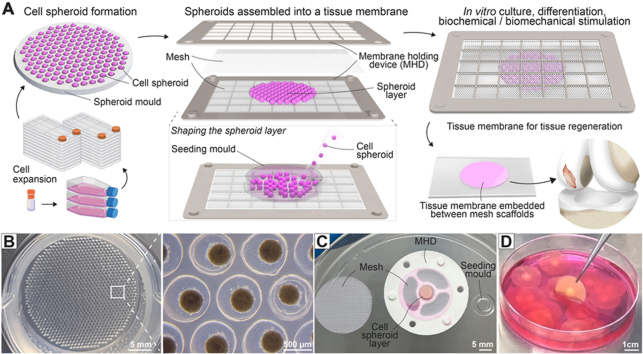

## 1 Introduction

Cell spheroids are widely used in tissue engineering due to their ability to mimic natural tissues' three-dimensional (3D) architecture. By promoting cell-cell interactions and enhancing extracellular matrix (ECM) production, spheroids create an environment conducive to the structural integrity and functionality of engineered tissues, thus reducing the need for artificial scaffolding [1]. Their successful utilization for making *in vitro* tissues, such as cartilage, bone, liver, cardiac, pancreatic, and neural tissues, has been well-documented in the literature [[2], [3], [4], [5], [6]]. Moreover, spheroids' scalability and tunability make them valuable in a range of research and therapeutic applications, including disease modelling, drug testing, and regenerative medicine, as extensively reviewed in the literature [7]. For instance, lung-derived spheroids formed from dissociated lung biopsies have been utilized to develop a pulmonary fibrosis model [8]. Similarly, human hepatocyte spheroids, which mimic liver's microarchitecture and exhibit some liver functions such as bile acid secretion and insulin response, have been employed to assess drugs' pharmacokinetics and toxicity [9]. In another example, mesenchymal stem cell (MSC) spheroids preconditioned for osteogenic differentiation were studied for bone regeneration in critical-sized femoral defects [10]. There are vast opportunities and commercial potentials for cell spheroid applications, fuelling significant interests in spheroid research [11].

Spheroids have the inherent ability to fuse together, making them the ideal building blocks for constructing larger and more intricate tissue structures. This fusion process is important for tissue development as it resembles the natural morphogenetic events during embryogenesis, where cellular aggregates coalesce to form complex organs and tissues [12]. The fusion enhances cell-cell interaction and deposition of ECM, thus improving the structural integrity of the tissue-engineered construct, enabling development of scaffold-free constructs [13]. However, when spheroids are put together, such as through bioprinting, they often merge into a single large mass due to a self-assembly process that is similar to the fusion of lipid droplets driven by surface tension [14]. This uncontrolled fusion not only disrupts the desired structural design but also impairs nutrient and waste diffusion, leading to cell death in the centre and causing significant gradients in growth factors, resulting in uneven tissue development.

Various tissue engineering strategies have been developed to control and organise the fusion of cell spheroids into macroscale tissue constructs [[15], [16], [17]]. For example, spheroids can be position in contact with each other within a mold made of non-adhesive materials like agarose or hyaluronic acid, which do not promote cell spreading, thereby facilitating the spheroid fusion. Commercial products are available that enable users to cast 3D molds to form cells or spheroids into microtissues of certain shape [18,19]. While this method is simple and convenient, it has been shown that the unconstrained spheroids will re-organise and contract due to cytoskeleton tension, resulting in structures that deviate from the intended mold shape [20,21]. To address this issue, macroporous scaffolds with lattice structures made of mechanically stable materials can be used to house the spheroids [22,23]. These scaffolds can be designed with highly interconnected macropores to facilitate nutrient diffusion throughout the tissue. However, this defeats the purpose of the scaffold-free tissue engineering approach. Moreover, achieving a balance between the scaffold's mechanical stability and degradability remains a challenge. Another method for controlling spheroid fusion involves using a needle array, where spheroids are skewered onto the needles using a bioprinter [24,25]. These needles are spaced closely to allow the spheroids to fuse into a 3D structure while supported by the array; once fused, the tissue can be released from the needle array. While successfully implemented, this method is not high throughput and requires complex equipment.

In this study, we present an innovative method to control the fusion of cell spheroids. Our approach involves confining the spheroids into a thin layer, supported by two mesh scaffolds, altogether held inside a gridded solid frame (patent-pending). This setup allows the spheroids to fuse into a membrane-like tissue, thin enough to maintain adequate medium diffusion. To demonstrate the efficacy of our method, we produced cartilage tissue membranes by assembling human mesenchymal stem cell (hMSC) spheroids in our membrane holding device (MHD) and differentiating the tissue construct in chondrogenic medium. We experimented with spheroid size, assembly timing, membrane thickness and monitored the culture for up to 14 days and characterized the tissue formed to evaluate the chondrogenic differentiation of the cells.

## 2 Results

### 2.1 Membrane holding device (MHD) restrains spheroid fusion into a membrane-like tissue

Cell spheroids, when left unrestrained, such as being placed in a well-shaped mold, will gradually fuse into a larger spherical-like mass regardless of the original shape of the mold [14]. The principle of our approach is to restrain the spheroids into a thin and uniform layer, resulting in a membrane-like tissue. This increases the surface area of the tissue compared to allowing the spheroids to self-assemble into a sphere-like structure, thus enhancing the diffusion of nutrients, growth factors, and oxygen to the tissue. Additionally, this method allows some control over the tissue thickness.

**Fig. 1:**
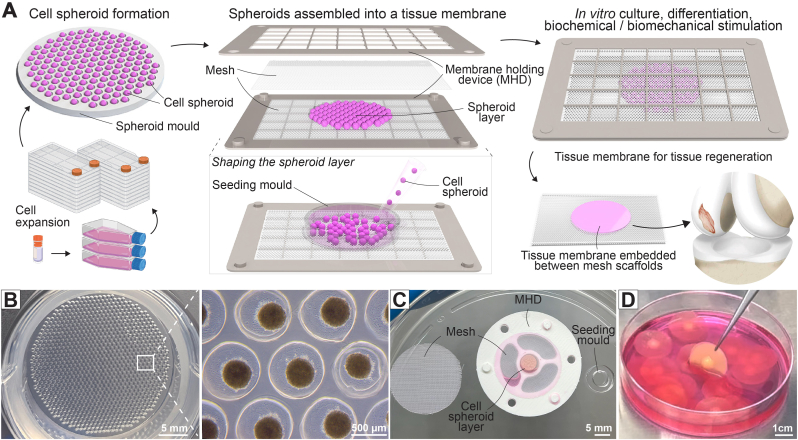
Method for making tissue membrane from cell spheroids. **(A)** Cells are expanded, made into spheroids and assembled between two mesh scaffolds, supported by a membrane holding device (MHD, patent-pending); the construct is cultured *in vitro* to stimulate spheroid fusion and differentiation into the target tissue type. **(B)** Agarose spheroid mold is used to make cell spheroids. **(C)** Forming a cell spheroid layer on the mesh using a seeding mold. **(D)** The final tissue membranes removed from the MHD after culture.

To restrain the cell spheroids, first we placed substrates on both sides of the spheroid layer, limiting fusion to a lateral direction. Next, we designed a MHD consisting of a solid frame that presses onto the mesh-spheroid-mesh assembly, maintaining the flatness of the membrane. The MHD is equipped with large grids to allow free medium passage through the construct (Fig. 1A). For optimum diffusion, we utilize thin mesh substrates with large mesh sizes, although the opening must be smaller than the spheroids to retain them. This mesh is in direct contact with the spheroids and can either be peeled off later or integrated into the final tissue membrane construct. The latter option provides additional mechanical strength to the tissue membrane, allowing it to be handled, wrapped, pulled, or sutured into defects during implantation.

To demonstrate the method, we used hMSC spheroids to construct a cartilage tissue membrane. The hMSC spheroids were generated on agarose spheroid molds, which yield 1000 spheroids per well of a 6-well plate (Fig. 1B). The spheroids were then harvested and transferred along with medium onto a mesh positioned over the bottom part of the MHD. A seeding mold was placed over the mesh to create a temporary well, into which the spheroids were pipetted. As medium drained from this mold, the spheroids settled onto the mesh to form a layer that maintained the shape and size of the mold (Fig. 1C, See also Supplemental Video 1**)**. A second mesh was then placed over the spheroid layer, followed by the top part of the MHD, and the entire assembly was secured with screws. The construct was cultured in chondrogenic medium to promote the fusion and differentiation of the hMSCs into a cartilage tissue membrane. At the end of the culture period, the MHD was opened to retrieve the mesh-spheroid-mesh construct (Fig. 1D, See also Supplemental Video 2**)**.

### 2.2 Size of spheroids affects hMSC chondrogenic differentiation

We explored how the size of hMSC spheroids affects chondrogenic differentiation when used to construct cartilage tissue membranes. Differentiation of hMSC *in vitro* is often inconsistent, largely due to donor variability. Traditional chondrogenic differentiation assay employs cell pellets with 250,000 cells to assess a donor's differentiation potential [26]. We hypothesized that varying the size of the spheroids could influence the chondrogenic differentiation capacity of donor MSC, due to smaller spheroids having better diffusion of medium and growth factors to all the cells in the spheroids.

**Fig. 2:**
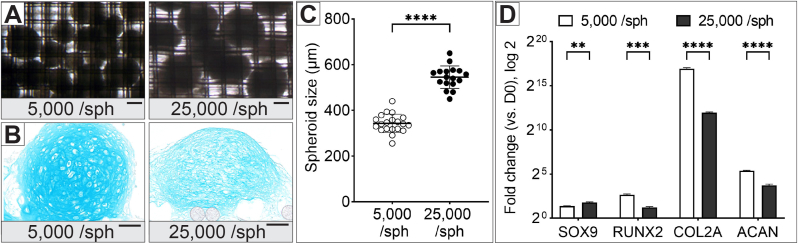
Impact of spheroid size on hMSC chondrogenic differentiation. **(A)** Brightfield images of hMSC spheroids containing 5,000 or 25,000 cells, assembled between two nylon meshes after 3 days of spheroid formation; scale bar = 200 μm. **(B)** Histological sections of 5,000 or 25,000 cell spheroids after 14 days in chondrogenic culture, stained with Alcian blue to visualise cartilage extracellular matrix (ECM); scale bar = 100 μm. **(C)** Scatter plot showing spheroid sizes of the 5,000-cell and 25,000-cell spheroids. **(D)** Gene expression analysis (RT-PCR) showing chondrogenic gene expression levels, with error bars representing the standard deviation of three technical replicates. Statistical significance is indicated as ∗∗p < 0.01, ∗∗∗p < 0.001, and ∗∗∗∗p < 0.0001.

Spheroids containing either 5,000 or 25,000 cells were produced using agarose molds and cultured for three days before being assembled between two nylon meshes secured by the MHD. At day 3, the 5,000-cell spheroids measured 300–400 μm, while the 25,000-cell spheroids were larger, measuring 500–600 μm (Fig. 2A and C). It was observed that spheroids left in the agarose molds for longer periods tended to float out of their wells and randomly fuse, resulting in non-homogeneous spheroid sizes. This issue is common and has led to the development of physical methods to keep spheroids separated within their wells, such as the microwell-mesh device by Futrega et al. [27]. In our approach, we aim to control the fusion of spheroids to form a membrane with a tunable shape and consistent thickness.

After 14 days in chondrogenic medium, histological analysis using Alcian blue staining revealed that the 5,000-cell spheroids exhibited a better cartilage morphology, characterized by an abundant, intensely stained ECM and round cell lacunae typical of cartilage tissue (Fig. 2B). In contrast, the 25,000-cell spheroids formed a more fibrous ECM with weaker staining, indicating less effective cartilage formation.

Gene expression analysis supported these observations, showing higher levels of early chondrogenic markers in the 5,000-cell spheroids (Fig. 2D). Specifically, RUNX2 expression was three times higher in the 5,000-cell spheroids (6.3-fold) compared to the 25,000-cell spheroids (2.3-fold). RUNX2 is a transcription factor involved in osteoblast differentiation and chondrocyte maturation, with increased expression linked to enhanced chondrocyte maturation and progression toward endochondral ossification [28]. Collagen type 2 (COL2A), the ECM protein of articular cartilage, was expressed 30 times higher in the smaller spheroids (125,000-fold vs. 4,000-fold), although both groups significantly upregulated COL2A compared to undifferentiated cells (day 0). Similarly, aggrecan (ACAN), a critical proteoglycan for cartilage's shock-absorbing function, showed 3 times higher expression in the smaller spheroids (40-fold vs. 13-fold). SOX9, a transcription factor for chondrocyte, was slightly elevated in the 25,000-cell spheroids (3.4-fold vs. 2.5-fold). Overall, the 5,000-cell spheroids demonstrated better chondrogenic differentiation, making them a more suitable building block for cartilage tissue membrane.

### 2.3 Effect of assembling timing to tissue membrane fusion and maturation

We investigated how the maturity of spheroid formation—1 day, 2 days, or 3 days—impacts their suitability for membrane assembly. At these early stages, the spheroids are newly formed, with minimal ECM deposition. These spheroids are primarily held together by cell-cell adhesion molecules, making them more fluidic and can easily fuse or be molded into various shapes.

**Fig. 3:**
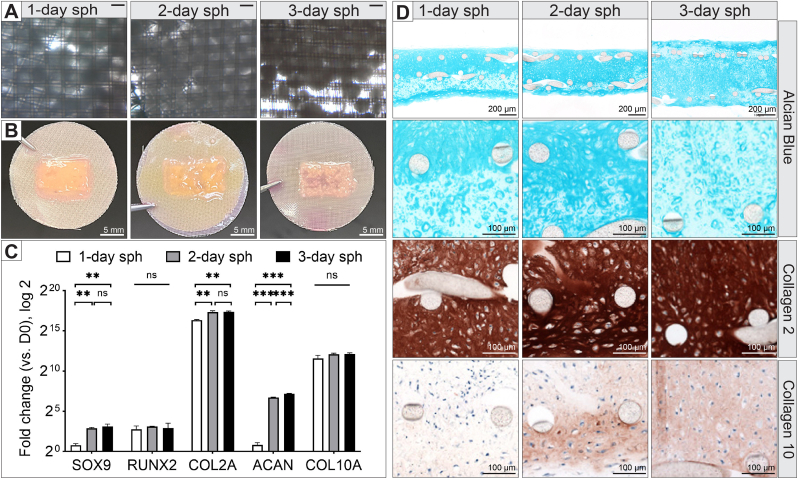
Impact of spheroid maturation timing on tissue membrane fusion and differentiation. **(A)** Brightfield images showing assemblies constructed from 1-day, 2-day, and 3-day spheroids on the day of assembly; scale bar = 200 μm. **(B)** Tissue membranes harvested from the MHD after 14 days of culture in chondrogenic medium; scale bar = 5 mm. **(C)** Gene expression analysis of the 14-day cultured tissue membranes, with error bars representing the standard deviation of three technical replicates. Statistical significance is indicated as ns: non-significant, ∗∗p < 0.01, and ∗∗∗p < 0.001. **(D)** Histological sections of the tissue membranes after 14 days, stained with Alcian blue and immunostained for collagen type 2 and 10.

We assembled the same number of spheroids (5,000 cells per spheroid), formed after 1, 2, or 3 days of seeding on the spheroid mold (referred to as 1-day, 2-day, or 3-day spheroids), onto the nylon mesh-MHD setup. On the day of assembly, the spheroids appeared similar, though the 3-day spheroids were slightly more distinct, with their spherical shape being more recognizable (Fig. 3A). After 14 days of culture in chondrogenic medium, the 1-day spheroids formed the smoothest tissue membrane. In contrast, the 2-day and 3-day spheroids were less fused, resulting in gaps or areas lacking cells in the tissue membrane (Fig. 3B).

Gene expression analysis at day 14 indicated better chondrogenic differentiation in the 2-day and 3-day spheroids compared to the 1-day spheroids, as evidenced by higher expression of early chondrogenic markers (SOX9, COL2A, and ACAN) (Fig. 3C). No significant differences were observed in RUNX2 and COL10A expression across the groups. The most pronounced differences were in SOX9 and ACAN expressions, which were 3 times and 50 times higher in the 2-day and 3-day spheroids, respectively, compared to the 1-day spheroids.

Histologically, despite having the same number of cells at the beginning and assembled on the same type of MHD, there were differences in the thickness of the tissue membranes (Fig. 3D). Specifically, the 3-day spheroids formed thicker tissues than the other two groups (750 μm vs. 550 μm). In all groups, cells were observed growing out of the nylon mesh and encapsulating the mesh struts. However, cells from the 3-day spheroid group seemed to proliferate more, pushing the two meshes further apart and overall forming a thicker tissue than intended. Alcian blue staining revealed patches of tissue with reduced staining. These areas exhibited larger cell lacunae and stained positive for collagen type 10, indicating hypertrophic cartilage tissue, a final stage in cartilage development where cells enlarge, undergo apoptosis, and release enzymes that degrade the ECM. Larger area of collage type 10 positive stain was observed in the 3-day spheroid group. Despite this, collagen type 2 immunostaining showed relatively even staining across the tissue in all groups, suggesting that collagen type 2 was not yet affected by the enzymatic activity of hypertrophic chondrocytes. Overall, this experiment demonstrates that the timing of spheroid maturation plays a critical role in tissue assembly, influencing both the fusion of spheroids and the differentiation outcomes in the engineered tissue.

### 2.4 Development of spheroid-derived cartilage tissue membrane over time

**Fig. 4:**
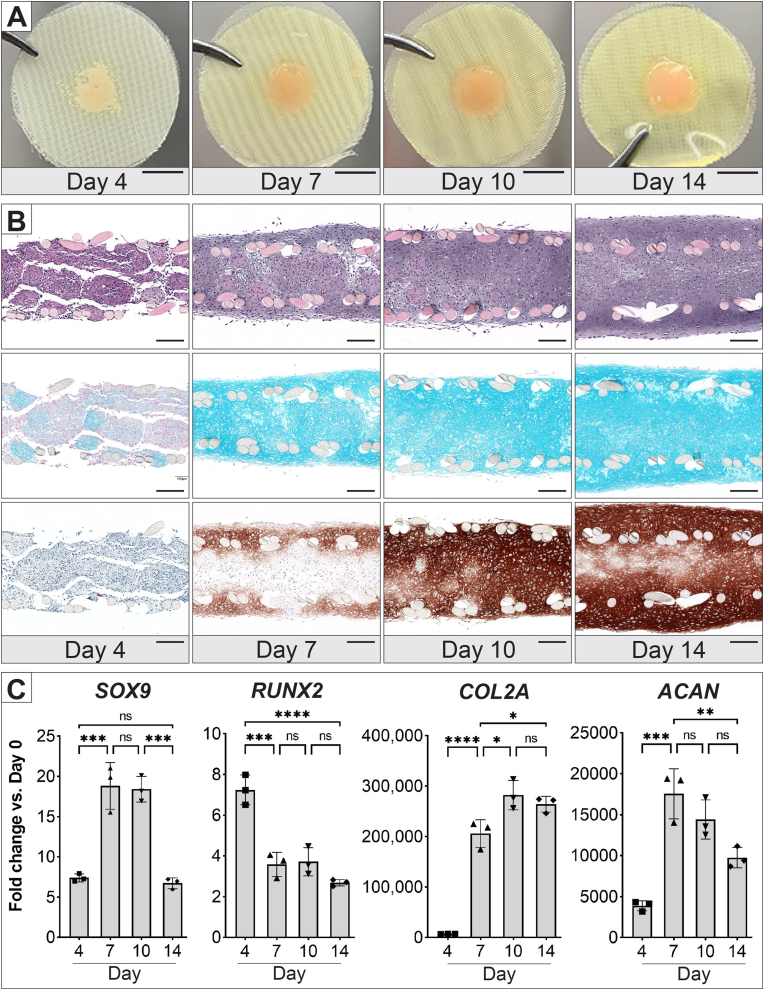
Fusion of the hMSC spheroids on membrane assembly over time. **(A)** Images of tissue membranes harvested at each time point; scale bar = 5 mm. **(B)** Representative histological sections of the tissue membranes at each time point, stained with Hematoxylin and Eosin (H&E, top), Alcian blue (middle), and immunostained for collagen type 2 (bottom); scale bar = 200 μm. **(C)** Gene expression analysis of tissue membranes cultured in chondrogenic medium over time, with error bars representing the standard deviation of three technical replicates. Statistical significance is indicated as ns: non-significant, ∗p < 0.05, ∗∗p < 0.01, ∗∗∗p < 0.001, and ∗∗∗∗p < 0.0001.

A time-course experiment was conducted to observe the fusion of hMSC spheroids and the differentiation of the tissue membrane over time. Spheroids were assembled on the nylon mesh-MHD setup after 3 days of formation and cultured in chondrogenic medium for 14 days. The membranes were harvested for analysis on days 4, 7, 10, and 14. The membranes' gross appearance showed progressive tissue growth, indicating cell proliferation happening during this time (Fig. 4A).

Histological cross-sections revealed the gradual fusion of spheroids over time. By day 4, many spheroids had begun to fuse, though some remained separated. The membrane assembly was 500–600 μm thick, with all spheroids contained within the nylon meshes. Alcian blue staining was visible in some spheroids, but collagen type 2 was not detected (Fig. 4B). By day 7, most spheroids had fused or were bridged by proliferating cells. Cells had started to migrate beyond the nylon mesh, contributing to increased tissue thickness. The entire tissue stained with Alcian blue, while collagen type 2 staining was observed primarily in the membrane's outer regions. At day 10, the ECM became more abundant and stained more intensely for both Alcian blue and collagen type 2. By day 14, the tissue membrane had thickened to approximately 800 μm, with about 40 % of the thickness extending beyond the mesh boundaries. The increasing distance between the two meshes suggested pressure from proliferating cells. Patches of fainter staining for both Alcian blue and collagen type 2 were also observed, indicating areas of hypertrophic chondrocytes.

Gene expression analysis at each time point showed dynamic changes in chondrogenic markers. SOX9 expression peaked at days 7 and 10 before decreasing by day 14, while RUNX2 peaked early at day 4 and declined thereafter. Collagen type 2 expression surged significantly from 4000-fold at day 4–200,000-fold at day 7, then remaining stable around 270,000-fold through day 14. Aggrecan expression peaked at 17,000-fold on day 7 before declining. Overall, collagen type 2 was the most strongly expressed protein throughout the culture period.

### 2.5 Proteome profiling of cartilage tissue membrane from hMSC spheroids

**Fig. 5:**
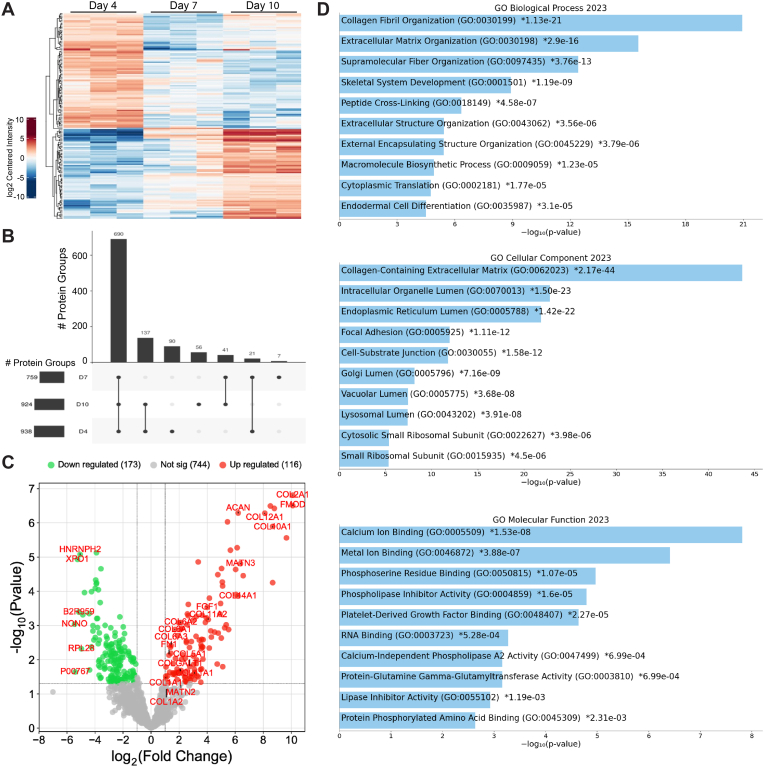
Characterization of protein expression in *vitro* cartilage development. **(A)** Heat-map illustrating the log2 centered intensity of proteins identified at Day 4, 7, and 10. The color gradient represents intensity levels, with blue indicating lower expression and red indicating higher expression, showing distinct clustering patterns over the time course. **(B)** Bar plot showing the number of protein groups identified at each condition: 938 at Day 4, 759 at Day 7, and 924 at Day 10, highlighting the variability in protein identification across different stages. **(C)** Volcano plot depicting differentially expressed proteins at Day 10 compared to Day 4. The x-axis represents log2 fold change (D10 vs D4) and the y-axis represents -log10 (p-value). Proteins significantly upregulated are shown in red, downregulated in green, and not significant in grey. Key proteins involved in cartilage development, such as COL2A1, COL10A1, CRTAP, COL11A2, DPT, COL11A1, COL5A1, COLGALT1, COL1A1, and ACAN, are labeled. **(D)** Gene Ontology (GO) pathways for differentially expressed genes in Day 10 compared to Day 4, categorized into Biological Processes, Cellular Components, and Molecular Functions related to cartilage development. The x-axis represents -log10 (p-value) for the enrichment of each pathway.

The proteomic analysis revealed significant variations in protein expression across the stages of cartilage development. Samples were collected on Days 4, 7, and 10 under controlled conditions. Cartilage tissues from each time point underwent rigorous sample preparation, followed by LC-MS/MS analysis, ensuring high-resolution and accurate protein identification. The heat-map (Fig. 5A) illustrates distinct clusters of proteins with varied expression levels at Day 4, Day 7, and Day 10, highlighting the dynamic nature of the proteome during this period. Clustering analysis underscores several proteins that exhibited substantial increases or decreases in expression at specific time points, indicating their pivotal roles in cartilage maturation.

The bar plot (Fig. 5B) shows the number of protein groups identified at each time point, with 938, 759, and 924 groups identified at Day 4, Day 7, and Day 10, respectively. This fluctuation emphasizes the dynamic shifts in the proteomic landscape during different stages of cartilage development. The volcano plot (Fig. 5C) presents the differentially expressed proteins at Day 10 compared to Day 4. There were 116 proteins significantly upregulated and 173 downregulated. Key proteins involved in cartilage development, including COL2A1 (Collagen Type II Alpha 1 Chain), COL10A1 (Collagen Type X Alpha 1 Chain), CRTAP (Cartilage Associated Protein), COL11A2 (Collagen Type XI Alpha 2 Chain), DPT (Dermatopontin), COL11A1 (Collagen Type XI Alpha 1 Chain), COL5A1 (Collagen Type V Alpha 1 Chain), COLGALT1 (Collagen Beta(1-O)Galactosyltransferase 1), COL1A1 (Collagen Type I Alpha 1 Chain) and ACAN (Aggrecan), showed substantial upregulation at Day 10, suggesting their critical roles in cartilage maturation and structural integrity. Additionally, proteins associated with ECM organization and cellular adhesion were notably upregulated, reflecting enhanced matrix remodeling and cellular interactions as cartilage matures.

Gene Ontology (GO) analysis (Fig. 5D) identified several pathways relevant to cartilage development. The Biological Processes category highlighted pathways such as collagen fibril organization (GO:0030199) and ECM organization (GO:0030198), underscoring the essential role of ECM in cartilage formation and maintenance. Cellular Components analysis revealed enrichment in collagen-containing ECM (GO:0062023) and intracellular organelle lumen (GO:0070013), emphasizing the localization and functional significance of these proteins within the cartilage ECM. Molecular Functions analysis indicated significant enrichment in calcium ion binding (GO:0005509) and metal ion binding (GO:0046872), crucial for the structural and functional properties of cartilage proteins.

The longitudinal data suggest a phased expression pattern, with early time points (Day 4 and Day 7) showing a high prevalence of proteins involved in cell proliferation and differentiation. In contrast, later time points (Day 10) exhibited upregulation of proteins associated with ECM assembly and maturation, reflecting a transition from proliferative to differentiation and maturation phases in cartilage development.

Our proteomic analysis supports the characterization findings by confirming the presence of key proteins and pathways associated with cartilage maturation and ECM organization. This correlation underscores the robustness of our method and reinforces the critical roles these proteins play in cartilage development.

### 2.6 Characterization of *in vitro* cartilage tissue membrane after 14 days

We evaluated the cartilage tissue membrane formed from hMSC spheroids after 14 days in chondrogenic medium on the MHD setup. To approximate the thickness of human articular cartilage, which exceeds 1 mm, we assembled 2,000 hMSC spheroids, each containing 5,000 cells, within a 5-mm diameter area on the nylon mesh—doubling the spheroid density used in prior experiments.

**Fig. 6:**
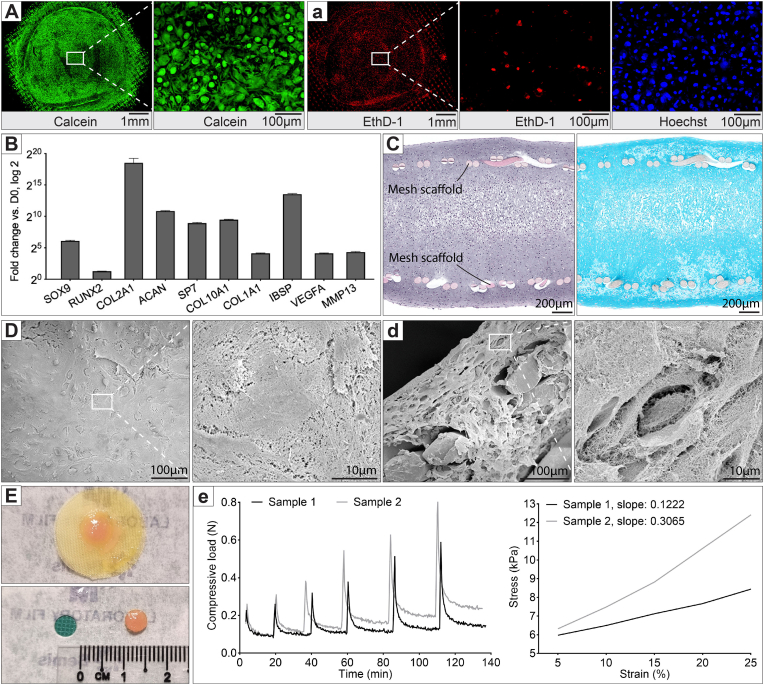
Characterization of cartilage tissue membrane from hMSC spheroids. **(A)** Confocal microscopy images of the 14-day chondrogenic tissue membrane stained with calcein (green) to visualize live cells, **(a)** ethidium homodimer (EthD-1, red) to visualize dead cells, and Hoechst stain for cell nuclei. **(B)** Gene expression analysis showing levels of early chondrogenic markers (SOX9, RUNX2, COL2A, ACAN), hypertrophic markers (SP7, COL10A1, MMP13), and bone-related markers (COL1A1, IBSP, VEGFA), with error bars representing the standard deviation of three technical replicates. **(C)** Histological sections of the tissue membrane stained with H&E (left) and Alcian blue (right). **(D)** Scanning electron microscopy (SEM) images of the tissue membrane surface and **(d)** cross-sections of the membrane. **(E)** The tissue membrane after being punched out for mechanical testing, and **(e)** force-time graph generated during unconfined equilibrium compression test and stress-stain curve generated from the stress relaxation phase.

First, we assessed cell viability within the tissue membrane using a live/dead staining assay combined with confocal microscopy, allowing visualisation up to 200–300 μm into the tissue. The 14-day cultured tissue membrane exhibited good cell viability at this depth, with the majority of cells stained with calcein dye, indicating they were alive (Fig. 6A, See also Supplemental Video 3**)**. Only a small number of cells was stained with ethidium homodimer (EthD-1), indicating dead cells (Fig. 6a).

Next, we performed gene expression analysis of the tissue membranes. In addition to high expression of early chondrogenic markers (SOX9, RUNX2, COL2A, and ACAN), we observed relatively high expression of hypertrophic markers (SP7, COL10A1, MMP13) and bone markers (COL1A1, IBSP, VEGFA), suggesting that the tissue had not only differentiated into cartilage but also progressed into the hypertrophic stage and possibly early bone development (Fig. 6B). Histological analysis of cross-sections revealed a tissue thickness of approximately 1.6 mm. About one-third of the tissue on either side of the membrane appeared to have developed into cartilage-like tissue, with abundant ECM staining positive for Alcian blue, while the middle third showed signs of necrosis, suggesting insufficient medium diffusion for this level of thickness (Fig. 6C). Scanning electron microscopy (SEM) was used to study the microstructure of the tissue. The surface of the tissue membrane was relatively smooth and covered with dense ECM (Fig. 6D).

Finally, we evaluated the mechanical properties of the *in vitro*-developed cartilage tissue membrane using mechanical compression testing. The nylon mesh remained integrated with the tissue, and the entire construct was punched out using a 5-mm biopsy punch (Fig. 6E). Using an equilibrium compression method, where the sample was submerged in saline and incrementally compressed with relaxation allowed after each compression, we measured equilibrium moduli of 0.12 and 0.3 MPa for our two samples (Fig. 6e). Overall, these results indicate that while the tissue membrane developed some characteristics of cartilage tissue, the increased thickness led to a necrotic band and potentially non-homogeneous differentiation within the tissue construct.

## 3 Discussion

In this study, we explored a novel method for assembling cell spheroids by arranging them in a thin layer supported by two mesh scaffolds and a solid frame, thus restraining the spheroids fusion into a membrane-like tissue. Our experiments with hMSC spheroids differentiated into cartilage tissue membranes demonstrated the validity of this approach. The resultant membrane-like cartilage tissue exhibited similar characteristics of cartilage tissue, including a smooth surface, abundant ECM that stained positively for Alcian blue and collagen type II, and upregulated expression of chondrogenic genes.

There are several challenges in bottom-up tissue engineering using cell spheroids. First, the production of spheroids, with methods such as hanging drops, non-adherent well plates, microwell arrays, suspension cultures (e.g., spinner flasks, magnetic levitation), microfluidic chips, and porous scaffolds; each has advantages and limitations [15,29]. In this study, we utilized the microwell array approach, using agarose microwell molds produced in-house from a negative silicone mold. This allows us to produce thousands of spheroids per well in a 6-well plate, achieving reasonably uniform sizes. To scale this process for GMP manufacturing, the production protocol must ensure consistency of spheroids for every batch, as well as minimizing carry-over contaminants to the next step [30].

Once spheroids are formed, the next challenge is to prevent them from randomly fusing together because random fusion result in non-uniform spheroid sizes, affecting the quality of the assembly. One solution is to separate spheroids into individual wells or design microwell arrays with deeper wells; however, these can complicate spheroid retrieval. For instance, Futrega et al. addressed this issue by designing a microwell-mesh system to trap spheroids in place [27]. In our experiments, we used a combination of sufficiently deep microwell arrays (about 1 mm) and a short harvest time (1–3 days post-spheroid formation) to minimize spheroid movement while maintaining ease of retrieval.

The final challenge is using spheroids to form larger tissue assembly. One straightforward approach is to position spheroids together in an unconstrained mold. For instance, Czajka et al. utilized a rectangular agarose mold, placing 8,000-cell spheroids in direct contact. They reported that the resulting sheet-like tissues tended to roll inward from the edges, forming irregular structures due to cytoskeletal tension [20]. In another example, Kim et al. produced cardiac spheroids containing 0.6x10^6^ or 1.4x10^6^ cells and positioned five spheroids in contact with each other in a longitudinal agarose mold to promote fusion into elongated microtissues [31]. The author demonstrated the formation of an elongated cardiac microtissue with action potential propagation across the tissue. However, the study did not report results beyond 3 days of culture, posing questions about the long-term stability and structural changes of the microtissue.

The molds used in the above-mentioned studies share a common feature: they are made from hydrogel materials, specifically agarose, which prevent cells from spreading on the mold surface and support medium diffusion. However, these molds do not precisely fit the spheroid assembly and lack the capacity to apply physical forces for aligning or restraining the spheroids. As a result, they are less effective in controlling the size and shape of the tissue assembly. Consequently, tissues formed in such unconstrained molds often fail to achieve the desired structure and design needed for functional tissue constructs.

The key advantages of our spheroid-membrane assembling method is that the porous mesh provides strong yet flexible restraining support, which is reinforced by an external solid frame that ensures a fixed spacing to keep the spheroid layers confined. Moreover, the mesh can be used to apply mechanical stimulation, such as cyclical stretching, if needed for development of tissue for example muscle tissue. This method is highly scalable, allowing for the production of large tissue membrane without requiring complex designs or specialized equipment.

We opted for a commercially available nylon mesh due to its ready availability in various pore sizes. nylon, a polyamide, is widely used in medical implants because of its biocompatibility and high tensile strength, making it suitable for applications such as high-tension non-absorbable sutures or vascular grafts [32]. Additionally, nylon is a bioinert material, which indirectly encourages cell aggregation and fusion rather than spreading to the mesh surface. This characteristic is important for scaffold-free tissue engineering strategies, which depend on the ECM produced by cells to serve as the tissue's natural scaffold, binding the cells together and forming the tissue structure [33]. However, since nylon is non-resorbable, it may not be ideal for applications requiring scaffold resorption. For such applications, biodegradable materials like polylactic acid (PLA), polyglycolic acid (PGA), and polycaprolactone (PCL) are preferable; and meshes often needed to be fabricated in-house from these materials due to the lack of off-the-shelf options.

We selected nylon mesh with a pore size of 150 μm by 200 μm to effectively retain the cell spheroids, which typically exceed 150 μm in diameter. This large pore size ensures unimpeded medium flow. However, our time-point experiments revealed that while the spheroids are initially contained within the mesh assembly, they progressively migrate across the mesh, leading to formation of a thicker tissue that eventually impedes medium diffusion. To address this issue, using meshes with micropores could help maintain the desired tissue membrane thickness by preventing cells from crossing the mesh. This approach could also be advantageous in allogeneic implantation, where the mesh acts as a barrier to shield the cells from the immune system. A relevant example is provided by Lathuilière et al., who developed an implantable cell encapsulation system using permeable polypropylene membranes with 0.45 μm pore sizes sealed to a solid frame [34]. This device, filled with a single cell suspension in a hydrogel solution, demonstrated long-term cell viability *in vivo* with minimal inflammatory response and dense neovascularization in the surrounding host tissue. However, a limitation of such a system is the reduced diffusion capacity due to the less porous membrane. While this device may appear similar to our MHD, their primary focus is on evading the immune system *in vivo*, whereas our objective is to assemble cell spheroids into a membrane tissue *in vitro*.

The diffusion limit for *in vitro*-cultured tissue typically ranges from 100 to 200 μm, depending on the tissue type. For tissues with high metabolic activity, such as cardiac, liver, or neural tissues, the diffusion limit is generally closer to 100 μm. In contrast, tissues with lower metabolic demands, like cartilage, have lower oxygen requirements, allowing their diffusion limit *in vitro* to extend closer to 200 μm or more [35]. Theoretically, with our membrane assembly having both sides exposed to the medium, it could support a tissue thickness of up to 400 μm. In our experiments, we produced tissue membranes with thicknesses ranging from 500 μm to 1600 μm. It became evident that as the membrane become denser due to ECM production, or as its thickness increased due to cell proliferation, the tissue morphology became less homogeneous, and necrotic bands formed. This is due to the increased tissue density impeding diffusion. To overcome this thickness limitation while maintaining tissue viability, an active medium perfusion mechanism, potentially combined with mechanical stimulation, could be applied. This approach would more closely replicate the *in vivo* environment. For example, in articular cartilage, cyclic compression and decompression facilitates the exchange of synovial fluid and supports cartilage thickness in the body up to 6 mm [36].

In our experiment, the cartilage membrane was assembled from bone marrow-derived hMSC spheroids induced with transforming growth factor beta 1 (TGF- β1). The results showed significant expression of hypertrophic markers at day 14 and large area of collagen type 10 stain, indicating that the tissue was undergoing endochondral ossification transition. Chondrogenic differentiation using a single factor like TGF- β1 are known to cause hypertrophy in cartilage tissue [37]. In this study, our primary focus was not on addressing this issue but on demonstrate the spheroid assembly method. For cartilage tissue engineering, various strategies such as the use of hypoxia and mechanical stimulation can be employed to reduce hypertrophic development [38].

It is also worth noting that other methods, such as 3D cell sheets, can also result in the formation of tissue membranes. However, 3D cell sheets are fundamentally different from spheroid cultures, even if the final construct achieves the same thickness. Cell-sheet-based structures are created by stacking monolayers of adherent cells that spread on a flat substrate. The cell sheets are detached intact using mechanisms such as temperature-responsive, photo-responsive, or pH-responsive method and then stacked to form a thicker construct [39]. While 3D cell sheets have unique applications in tissue engineering, they differ significantly from spheroid-based tissues [40]. The spreading of cells in monolayers and the stiffness of the substrate can profoundly influence cellular programming through mechanotransduction, affecting processes such as stem cell differentiation [41]. This behaviour is distinct from the cell aggregation in spheroids, which is crucial for initiating other biological processes [42].

In conclusion, this study introduces a novel platform technology for assembling cell spheroids into membrane-like tissue using a mesh-supported framework. The simplicity and modularity of this approach make it a promising strategy for various tissue engineering applications. However, challenges such as achieving consistent tissue morphology and preventing necrosis in thicker constructs indicate the need for further development.

## CRediT authorship contribution statement

**Quang Bach Le:** Writing – review & editing, Writing – original draft, Visualization, Validation, Supervision, Software, Resources, Project administration, Methodology, Investigation, Funding acquisition, Formal analysis, Data curation, Conceptualization. **Hariharan Ezhilarasu:** Writing – review & editing, Writing – original draft, Visualization, Validation, Project administration, Methodology, Investigation, Conceptualization. **Weng Wan Chan:** Writing – review & editing, Writing – original draft, Visualization, Supervision, Resources, Methodology, Investigation, Formal analysis, Conceptualization. **Alok Tanala Patra:** Data curation, Formal analysis, Investigation, Methodology, Resources, Software, Validation, Visualization, Writing – original draft, Writing – review & editing. **Priya Murugan:** Writing – original draft, Resources, Methodology, Data curation, Conceptualization. **Shashaank Abhinav Venkatesh:** Methodology, Investigation, Data curation, Conceptualization. **Yean Kai Tay:** Methodology, Formal analysis, Data curation, Conceptualization. **Shin Ru Lim:** Methodology, Investigation, Data curation, Conceptualization. **Ahmad Amirul Abdul Rahim:** Methodology, Conceptualization. **Jia Sheng Zach Lee:** Methodology, Conceptualization. **Xuezhi Bi:** Conceptualization, Funding acquisition, Project administration, Resources, Software, Supervision, Writing – review & editing. **Deepak Choudhury:** Writing – review & editing, Writing – original draft, Visualization, Validation, Supervision, Software, Resources, Project administration, Methodology, Investigation, Funding acquisition, Formal analysis, Data curation, Conceptualization.

## Declaration of generative AI and AI-assisted technologies in the writing process

During the preparation of this work the authors used ChatGPT-4o to enhance the language and readability of the text. After using this tool, the authors reviewed and edited the content as needed and takes full responsibility for the content of the publication.

## Declaration of competing interest

The method for constructing tissue membranes from cell spheroids, along with the membrane holding device, is included in a patent application, with Q.B.L., D.C., and W.W.C. listed as inventors. The authors declare no additional competing interests.

## Data Availability

Data will be made available on request.
