# Supplementary material for: A platform for Bioengineering Tissue Membranes from cell spheroids
Source: Mater Today Bio. 2025 Jan 31;31:101526. doi: 10.1016/j.mtbio.2025.101526 (PMC11869014; doi:10.1016/j.mtbio.2025.101526)
Supplement: Multimedia component 1 [file mmc1.docx]

**Supplemental Video 1 |** The process of seeding hMSC spheroids onto the initial Nylon mesh layer during the assembly of the tissue membrane.

**Supplemental Video 2 |** Disassembling of the MHD setup and harvesting of the tissue membrane construct after the *in vitro* culture period.

**Supplemental Video 3 |** Three-dimensional rendered visualisation of stacked confocal images, showing live cells stained with Calcein within a 300 µm depth from the surface of the tissue membrane.

**Methods**

***Cell culture and spheroid formation***

Human bone marrow-derived mesenchymal stem cells (hMSCs) (Lonza, USA) were expanded in a growth medium composed of low-glucose DMEM, 1% penicillin-streptomycin, 10% FBS, 5 µL/mL heparin, and 10 ng/mL FGF-1 (PeproTech, USA). All media and supplements were purchased from ThermoFisher Scientific (USA) unless otherwise stated. The cells were cultured in tissue culture-treated flasks at 37°C with 5% CO2 and 90% humidity, with medium changes every 2–3 days. For larger-scale expansion, HyperFlask M (Corning, USA) vessels were utilised to minimise handling. The cells were harvested at passage 6 for spheroid formation using agarose spheroid molds.

Agarose spheroid mold plates with microwells (700 µm in diameter and depth, approximately 1,000 microwells per well in a 6-well plate) were prepared aseptically by transferring a pattern from a silicone mold (produced in-house) into molten 1.5% agarose within a 6-well plate. The molten agarose was allowed to cool for 15 minutes, after which the silicone mold was carefully removed to create an array of microwells in the well plate. The agarose spheroid molds were preconditioned in chondrogenic medium for 24 hours at 4°C before spheroid formation.

Following cell expansion, hMSCs were harvested and resuspended in chondrogenic medium composed of high-glucose DMEM, 1% penicillin-streptomycin, 1% ITS-X, 200 µM 2-phospho-L-ascorbic acid trisodium salt (Sigma-Aldrich, USA), 40 µg/mL L-proline (Sigma-Aldrich, USA), 100 nM dexamethasone (Sigma-Aldrich, USA), and 10 ng/mL TGF-β1 (PeproTech, USA). The appropriate number of cells was pipetted dropwise onto the agarose spheroid molds, typically 5 million cells per well, resulting in 5,000 cells per microwell. The plate was centrifuged at 150 x g for 2 minutes to ensure even distribution of cells into the microwells, and then carefully transferred to an incubator to facilitate spheroid.

***Spheroid harvesting and assembly onto the membrane assembly***

Spheroids were harvested 3 days after seeding on the agarose molds, except in experiments focused on maturation timing. Harvesting was done by repeatedly flushing the wells with a 1 mL pipette to collect all spheroids from each well into a separate tube, ensuring accurate tracking of spheroid numbers (about 1,000 spheroids per tube). All components used in membrane assembly, including Nylon meshes (Simport, Canada), Nylon screws (Tr Fastening, UK), silicone seeding molds (fabricated in-house from polydimethylsiloxane–Dow, USA), and necessary tools (screwdrivers, forceps, spatulas), were sterilised by autoclaving. The membrane holding device (MHD) was 3D printed using polylactic acid material and sterilised by soaking in 70% ethanol for 24 hours.

Spheroids were transferred onto the Nylon mesh using a 1 mL pipette, with the silicone seeding mold serving as a temporary well to shape and size the spheroid layer (See also **Supplemental Video 1**). Once the spheroids settled on the Nylon mesh, the seeding mold was removed. If needed, the spheroid layer could be adjusted with a small spatula to ensure even distribution. The spheroid layer was then covered with a second layer of Nylon mesh, and the top part of the MHD was positioned and secured with screws. The entire construct was submerged in chondrogenic medium and cultured for a total of 14 days. To harvest the tissue membrane, the MHD was disassembled by removing the screws and the top part. The tissue membrane, now consisting of the tissue embedded between two Nylon meshes, was then removed for further analysis (See also **Supplemental Video 2**).

***Reverse-transcription polymerase chain reaction (RT-PCR)***

Tissue membrane samples were collected at various time points and immediately frozen at -80°C until RNA isolation. RNA was extracted using the PureLink RNA Mini Kit (ThermoFisher Scientific, USA). Briefly, lysis buffer containing 2-mercaptoethanol was added to the tissue membrane placed in a small petri dish. The Nylon meshes were then peeled off to harvest the tissue; any residual tissue attached to the mesh was scraped off using a spatula. The tissue in lysis buffer was homogenized at maximum speed using a homogeniser. The homogenate was then centrifuged, and the supernatant containing the RNA was mixed with 70% ethanol (1:1 v/v) before being loaded onto spin columns. The RNA was washed with the kit’s washing buffer and eluted in RNase-free water.

The RNA concentration was measured using a NanoDrop spectrophotometer (ThermoFisher Scientific, USA). Reverse transcription PCR (RT-PCR) was performed with 10 ng of RNA on a CFX96 system (BioRad, USA), using the iTaq Universal One-Step Kit (BioRad, USA) and TaqMan assays (ThermoFisher Scientific, USA) specific to the target genes. The assays included reference gene ACTB (Hs01060665_g1), COL2A1 (Hs00264051_m1), ACAN (Hs00153936_m1), SOX9 (Hs00165814_m1), RUNX2 (Hs01047973_m1), COL10A1 (Hs00166657_m1), MMP13 (Hs00942584_m1), SP7 (Hs01866874_s1), VEGFA (Hs00900055_m1), COL1A1 (Hs00164004_m1), COL10A (Hs00166657_m1) and IBSP (Hs00913377_m1). Fold changes were calculated using the ΔΔCt method, with ACTB as the reference gene. Gene expression levels were compared to day 0 expression, which corresponds to hMSCs harvested after cell expansion and before exposure to chondrogenic medium. The data are presented as the log2 of the fold change relative to day 0 expression.

***Sample preparation and LC-MS/MS analysis***

Proteins were extracted from chondrocyte tissue using a lysis buffer containing 6 M urea, 1% SDS, and 50 mM ammonium bicarbonate (ABC). Tissue membrane samples were scraped off from the Nylon meshes in the presence of lysis buffer and homogenised for 10 minutes using a homogeniser (CAT X1000) at maximum speed, with tube kept on ice. The lysate was centrifuged at 15,000 x g for 20 minutes to remove cellular debris. Protein concentrations were determined using the Bradford assay. A total of 100 µg of protein was reduced, alkylated, and digested with trypsin at an enzyme-to-substrate ratio of 1:25 for 2 hours at 42°C on an S-Trap mini spin column (ProtiFi), following the manufacturer’s instructions. The eluted peptides were dried under vacuum using a speed-vac and further purified using an MCX in-house filter.

Peptides were reconstituted in 100 µL of a solution containing 2% acetonitrile (ACN) and 0.5% trifluoroacetic acid (TFA), and vortexed for 15 minutes to ensure proper solubilization. Prior to loading the samples onto the filter, it was conditioned sequentially with 50 µL of 100% ACN, followed by MilliQ water and 0.1% TFA, with each step centrifuged at 1,700 x g for 1 minute. The peptide samples (100 µL) were loaded onto the filter and centrifuged at 800 x g for 2 minutes. The flow-through was reloaded onto the filter to maximize peptide recovery, and the process was repeated. The filter was washed with 50 µL of 0.1% TFA in 2% ACN and centrifuged at 1,200 x g for 3 minutes, repeated twice.

Elution of peptides was performed with 20 µL of 50% ACN and 5% NH4OH, and centrifuged at 1,200 x g for 3 minutes, repeated thrice, yielding a total elution volume of 60 µL. The eluted peptides were dried using a speed-vac and reconstituted in a loading buffer containing 1% formic acid in 2% ACN for LC-MS/MS analysis.

Peptide analysis was conducted using a Thermo Eclipse Mass Spectrometer coupled with a C18 reverse-phase LC system. The mobile phase consisted of 0.1% formic acid in water (Solvent A) and 0.1% formic acid in acetonitrile (Solvent B). A gradient of 5-35% Solvent B was applied over 60 minutes. The mass spectrometer was operated in Data-Dependent Acquisition (DDA) mode with an MS1 scan range of 350-1,500 m/z at a resolution of 120,000. The 20 most intense ions were selected for MS/MS fragmentation using Higher-energy Collisional Dissociation (HCD) with a normalized collision energy of 30.

Data were acquired in DDA mode and processed using FragPipe, incorporating MSFragger and Philosopher software. Raw data were converted to mzML format, and protein identification was performed against the human UniProt reference proteome database (downloaded in September 2024). Search parameters were set as previously described [43, 44]. Peptide and protein validation was carried out using PeptideProphet and ProteinProphet, with label-free quantification achieved using IonQuant. Statistical and biological significance was inferred from the identified peptides and proteins, allowing for comprehensive data interpretation.

***Histological analysis***

Tissue membrane samples were fixed in 10% (v/v) neutral buffered formalin for 24 hours, then dehydrated, paraffin embedded, and sectioned longitudinally (7 µm thickness) from the middle of the membrane. Sections were stained with hematoxylin and eosin (H&E) to assess general morphology, and with Alcian blue (pH 1.0) to detect sulfated glycosaminoglycans, which are characteristic of cartilage tissue. Immunohistochemical staining for collagen type 2 (1:100, clone 6B3, ThermoFisher, USA) and collagen type 10 (1:100, clone X53, ThermoFisher, USA) was performed using the Bond Refine Detection Kit (DS9800) and the Bond III staining system (Leica, Germany). The sections were then scanned using a Carl Zeiss AxioScan 7 system (Zeiss, Germany) with a 20x objectives and subsequently processed with Zen Lite Microscopy suite software (v3.10, Zeiss, Germany).

***Live dead staining and confocal imaging***

The tissue membrane was washed with PBS and stained using the Live/Dead staining kit (ThermoFisher Scientific, USA), which includes calcein AM and ethidium homodimer-1 for distinguishing live from dead cells. A working solution was prepared containing 2 µM calcein AM, 4 µM ethidium homodimer-1, and Hoechst 33342 (ThermoFisher Scientific, USA) for nuclear staining in PBS. The samples were incubated with this solution at 37°C for 30 minutes, followed by a PBS wash to remove excess dye. The stained cells were visualised using a Nikon A1 confocal microscope, and the images and rendered videos were processed with NIS-Elements Imaging software (v5.21, Nikon, Japan).

***Scanning electron microscopy (SEM)***

Tissue membrane samples were harvested, rinsed with PBS, and fixed overnight in 10% (v/v) neutral buffered formalin. After fixation, samples were washed three times with PBS and then dehydrated through a graded ethanol series (70% to 100%) to remove water and prevent shrinkage of the sponges during imaging. The dehydrated samples were further processed using a critical point dryer (Leica CPD300) to remove the solvent while preserving the scaffold structure. The dried scaffolds were mounted on SEM studs with conductive adhesive carbon tape to prevent charging during imaging, and then coated with a 15 nm layer of platinum using a JSC-1200 fine coater (JEOL, Japan). The coated scaffolds were subsequently analysed using a field emission scanning electron microscope (FESEM) (JSM-6701F, JEOL, USA).

***Mechanical test***

The equilibrium modulus of the samples was determined using an Instron mechanical tester (Instron 5548). Samples were prepared by punching 5 mm biopsies and immersing them in PBS for 30 minutes. Sample height was measured under a pre-load of 0.1 N, ensuring the change in force was less than 0.01 N per minute. Compression testing was conducted from 0% to 30% strain in 5% increments, at a rate of 0.01 mm/min between intervals. At each predetermined interval, samples were held to achieve equilibrium stress, defined as a change in force of less than 0.098 N per minute. The stress-strain curve was plotted from 0% to 25% strain, and the equilibrium modulus was calculated from the linear region of the stress-strain curve.
